# Phytotherapy Might Have a Role in Reducing Unnecessary Prostate Biopsies: Results from an Exploratory, Randomized Controlled Trial of Two Different Phytotherapeutic Agents

**DOI:** 10.3390/clinpract14010016

**Published:** 2024-01-23

**Authors:** Tommaso Cai, Irene Tamanini, Marco Puglisi, Leonardo Bizzotto, Michele Rizzo, Giovanni Liguori, Luca Gallelli, Alessandro Palmieri, Truls E. Bjerklund Johansen

**Affiliations:** 1 Department of Urology, Santa Chiara Regional Hospital, 38122 Trento, Italy; irene.tamanini@apss.tn.it (I.T.); marco.puglisi@apss.tn.it (M.P.); 2 Institute of Clinical Medicine, University of Oslo, 0313 Oslo, Norway; t.e.b.johansen@medisin.uio.no; 3 Centro Servizi Sanitari (CST), 38121 Trento, Italy; leonardobizzotto86@gmail.com; 4 Department of Urology, University of Trieste, 34127 Trieste, Italy; michele.rizzo@asugi.sanita.fvg.it (M.R.); gliguori@units.it (G.L.); 5 Department of Health Science, School of Medicine, University of Catanzaro, 88100 Catanzaro, Italy; gallelli@unicz.it; 6 Department of Urology, University of Naples Federico II, 80138 Naples, Italy; info@alessandropalmieri.it; 7 Department of Urology, Oslo University Hospital, 0450 Oslo, Norway; 8 Institute of Clinical Medicine, University of Aarhus, 8000 Aarhus, Denmark

**Keywords:** prostate-specific antigen, prostate cancer, diagnosis, inflammation, phytotherapy

## Abstract

Background: We aimed to evaluate the impact of two different phytotherapeutic agents on decision making regarding prostate biopsy for patients with higher-than-normal prostate-specific antigen (PSA) levels. Methods: From June 2022 to May 2023, all patients attending two urological institutions due to higher-than-normal PSA levels were randomized to receive either oral capsules of Curcuma Longa, Boswellia, Pinus pinaster and Urtica dioica (Group A) or Serenoa Repens 320 mg (Group B) for 3 months. At the follow-up visit after 3 months, all patients underwent PSA tests and multiparametric magnetic resonance imaging (mpMRI). Results: In the per-protocol analysis, data from 66 patients in Group A and 76 in Group B were analyzed. Fifty patients in Group A (75.7%) showed a significant reduction in total PSA compared to forty-nine in Group B (64.4%) (*p* < 0.001). Twenty-eight patients had PI-RADS III or higher in mpMRI: twelve in Group A and fourteen in Group B. Twenty-three patients (34.8%) in Group A and fifty-nine (77.6%) in Group B (*p* < 0.001) underwent prostate biopsy based on the mpMRI findings and PSA values. Three patients in Group A showed a significant reduction in total PSA values while having positive mpMRI findings (6%) compared with nine in Group B (19.5%) (*p* < 0.001). On the contrary, 7 patients in Group A did not show significant reduction in total PSA values and had negative mpMRI findings (43%) compared to 22 in Group B (81.4%) (*p* < 0.001). Conclusions: In conclusion, a three-month course of a combination of Curcuma Longa, Boswellia, Pinus pinaster and Urtica dioica seems to be an interesting tool to avoid unnecessary prostate biopsies among men with higher-than-normal PSA levels.

## 1. Introduction

Prostate cancer represents the most common malignancy in men today, accounting for 23.2% of all new cancer cases in European countries, with high costs associated to cancer treatment and the management of complications [1]. An early diagnosis is important for improving survival and reducing complication rates and relevant costs [2,3]. Even though the diagnostic accuracy and specificity of an elevated serum prostate-specific antigen (PSA) level is being questioned, PSA remains an important screening tool for prostate cancer and decision making before prostate biopsy [3]. Indeed, making decisions on prostate biopsies for men with elevated age-related PSA findings and normal digital rectal examinations (DREs) is one of most challenging issues in urology. Recently, however, multiparametric magnetic resonance imaging (mpMRI) is increasingly being used for detection, staging, targeted biopsies and treatment monitoring of prostate cancer [3,4,5]. Furthermore, prostate biopsy is associated with an elevated risk of infectious complications such as acute prostatitis, urinary tract infections and sepsis, which are arguments for avoiding unnecessary biopsies [6]. The situation caused a trend among urologists to prescribe antibiotics to see if an elevated PSA in a man with negative DRE findings might be due to infection. But Kayalı Y. et al. demonstrated that there is no significant difference in the diagnosis of cancer, regardless of any PSA decrease after antibiotic therapy, suggesting that the use of antibiotics to reduce high PSA levels in order to prevent unnecessary biopsies is not recommended [7]. In this scenario, any therapeutic agent that is able to improve the diagnostic utility of PSA in the decision making before prostate biopsy might help reduce the number of unnecessary biopsies. Moreover, in the era of antimicrobial stewardship, all use of unnecessary antibiotics should be avoided and an antimicrobial-sparing approach should be preferred in the decision making before prostate biopsy [8]. Several phytotherapeutic and nutraceutical compounds show anti-inflammatory characteristics and are of interest in this field [9]. In the last years, the use of phytotherapeutic compounds such as curcuma longa, boswellia, urtica dioica, pinus pinaster has been explored in the management of urological diseases and has demonstrated minimal side-effects [10,11]. Based on the evidence above, we wanted to evaluate if a combination of Curcuma Longa, Boswellia, Pinus pinaster and Urtica dioica (Prostaflog^®^) might have a role in decision making before prostate biopsy.

## 2. Materials and Methods

### 2.1. Study Design and Schedule

**Figure 1:**
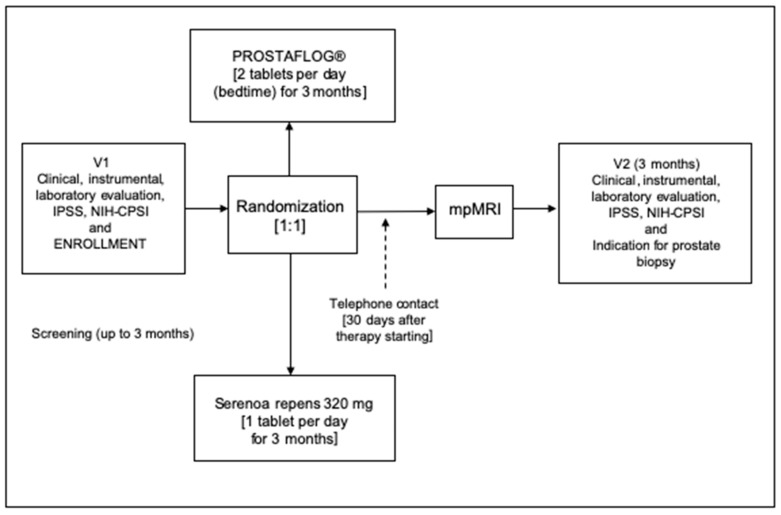
The figure shows the study schedule.

All consecutive patients with increased PSA values who attended two urological referral centers between June 2022 and May 2023 were enrolled in this exploratory, randomized, controlled phase III study. Patients in the treatment group received two capsules of Prostaflog^®^ at bedtime every day, while patients in the control group received 1 tablet of 320 mg Serenoa Repens daily. Patients in both groups underwent 3 months of treatment. Upon arrival at each center, all eligible patients gave written informed consent, provided the results of total and free/total PSA values, filled in baseline questionnaires and underwent urological examination with digital rectal examinations (DREs). All patients who met the inclusion criteria were randomized by using a computer-generated sequence of allocation. Patients were assigned to treatment groups according to a 1:1 randomization. Enrolled patients were not blinded. No placebo run-in period was considered necessary. All patients were contacted by telephone by a trialist on day 30 after initiation of therapy to ensure correct timing and dosing of treatment. At the end of the treatment, all patients were scheduled for mpMRI, a urological follow-up visit with new total and free/total PSA values and repeated baseline questionnaires. Prostate biopsies were performed on patients with suspected prostate cancer in mpMRI (PI-RADS III or higher) in cases in which total PSA had increased (>20% PSA level from the baseline) or there were suspicious findings in the DRE. The primary endpoint was the difference between groups in terms of change in PSA values between baseline and end of treatment (ΔPSA) and differences in histological findings. The study schedule is displayed in Figure 1.

### 2.2. Inclusion and Exclusion Criteria

We enrolled patients with increased PSA values, defined as a PSA value higher than 4.0 ng/mL at initial evaluation or a PSA value higher than 75% of the previous PSA evaluation, in line with Leslie et al. [12]. All patients with PSA higher than 10.0 ng/mL at initial evaluation were excluded. All patients with positive DREs at the initial evaluation were excluded from the present study. We excluded patients <18 years of age and patients with major concomitant diseases; a reported allergy to one or more components of the drugs; a history of chronic bacterial prostatitis or acute urinary tract infection; and recent transurethral diagnostics or treatment approaches, as well as patients receiving 5-alpha reductase inhibitor therapy due to benign prostatic hyperplasia and patients with indwelling catheters or intermittent catheterization. Moreover, we excluded all patients on active surveillance for prostate cancer or on hormonal treatment for prostate cancer. Furthermore, all patients on testosterone replacement therapy were excluded, too. All patients excluded from the present study due to PSA higher than 10.0 ng/mL or positive DREs at the initial evaluation underwent multiparametric magnetic resonance imaging and subsequent prostate biopsy with fusion biopsy technology in cases of suspected prostate lesions.

### 2.3. Laboratory Considerations

No central laboratory was considered necessary. All patients were asked to come to the urological centers with total PSA results and free/total PSA serum concentrations measured no more than 3 months earlier. PSA changes before and after treatment (ΔPSA) were evaluated separately for all patients and between the groups, according to PI-RADS II-IV lesions on mpMRI and findings of prostate cancer in biopsies. PSA reduction was defined as a reduction of 20% or more from the baseline, in line with De Nunzio et al. [13]. PSA testing was performed with automated immunoassays according to standard international recommendations [14]. PSA values were adjusted according to the age, in line with Heidegger I. et al. [15]. PSA testing was performed after a detailed discussion of the risks and benefits of prostate cancer screening activities and the study protocol aims.

### 2.4. Instrumental Considerations

Multiparametric magnetic resonance imaging was performed in line with international guidelines. mpMRI of the prostate combines the anatomic information from T1- and T2-weighted sequences with functional information from diffusion-weighted imaging and dynamic contrast enhancement. All mpMRIs were performed in line with the Prostate Imaging-Reporting and Data System (PI-RADS) v2.1 [16]. A centralized radiological evaluation was not considered necessary. An experienced radiologist in each center revised all mpMRIs according to the recommendations of the PI-RADS v2.1 manual [16]. Each prostatic lesion was assigned a score from 1 to 5 indicating the probability of clinically significant prostate cancer, as follows: PI-RADS 1: very low probability; PI-RADS 2: low probability; PI-RADS 3: intermediate probability; PI-RADS 4: high probability; PI-RADS 5: very high probability. All patients were asked to take 1 tablet of scopolamine butylbromide (BUSCOPAN^®^) 1 h before the examination in order to reduce the motion artifact from bowel peristalsis. No rectal enema was required before the examination. However, all patients were asked to evacuate the rectum, if possible, just before the mpMRI exam, according to the recommendations of the PI-RADS v2.1 manual [16]. Written informed consent to the radiologic procedure was required from all the patients, in line with the Italian bylaw and everyday clinical practice.

### 2.5. Questionnaires

The validated Italian versions of the International Prostatic Symptom Score (IPSS) [17] and NIH-Chronic Prostatitis Symptom Index (NIH-CPSI) [18] score were filled in by each patient on arrival to the urological outpatient clinic. The questionnaires were self-administered and only used for the initial clinical assessment at baseline as part of routine clinical practice. No follow-up data in terms of questionnaire results were reported.

### 2.6. Composition and Characterization of the Extracts Used

Each dose of Prostaflog^®^ contained 500 mg Curcuma longa, 300 mg boswellia, 240 mg urtica dioica and 200 mg pinus pinaster, as described in the manufacturer’s instructions (Naturneed, 62100, Macerata, Italy). All patients in the control group received 320 mg/die Serenoa Repens. Naturneed obtained the ISO 9001 certification, showing a robust quality-control process in pharmacological product production. The International Organization for Standardization-9001 certification reflects the quality of the pharmacological products, too.

### 2.7. Ethical and Statistical Considerations

This study was approved by the local ethics committee (approval protocol number 258, 2019), and it was conducted in compliance with the Institutional Review Board/Human Subjects Research Committee requirements and with the Declaration of Helsinki and the Guidelines for Good Clinical Trial Practice criteria. Written informed consent was obtained from all patients prior to treatment. According to the Italian Data Protection Authority recommendations (Law No. 675 of 31 December 1996; Legislative Decree No. 196 of 30 June 2003), all anamnestic, clinical and laboratory data containing sensitive information about patients were de-identified to ensure analysis of anonymous data only. The de-identification process was performed by non-medical staff by means of dedicated software. We stated that the study participation and the mpMRI performed within 3 months after referral would not cause delayed diagnosis for patients who turned out to have prostate cancer. Patients were informed accordingly. Randomization was based on a single sequence of random assignments (simple randomization) and performed using pseudo-random number generator software (Research Randomizer Version 4.0, Social Psychology Network, Wesleyan University, Middletown, CT, USA). All data were presented as means with median and IQR ranges. A χ^2^ test or Fisher exact test was used for categorical variables. Dependent, non-normally distributed variables were compared with Wilcoxon signed-rank test. Pearson’s correlation analysis was also used. Data obtained were analyzed by testing the difference between two proportions for incidence of prostate cancer in each group and evaluation of the ΔPSA. Sensitivity, specificity, positive and negative likelihood ratios and positive and negative predictive values were calculated. Statistical significance was achieved when *p* < 0.05. All reported *p*-values were two-sided. Statistical analyses were performed using SPSS software, version 22.0 (SPSS, Inc., Chicago, IL, USA) for Apple-Mac.

## 3. Results

### 3.1. Patient Populations

From an initial cohort of 182 patients attending our centers in the study enrollment period, 162 met the inclusion criteria and were enrolled. Patients were then randomly allocated, with 74 in Group A and 88 in Group B. Twenty patients were excluded from analysis due to missing data at the follow-up examination. In the per-protocol analysis, data from 66 patients in the Prostaflog^®^ group and 76 in the Serenoa Repens group were analyzed. Table 1 shows all demographic, anamnestic, clinical and laboratory data at enrollment.

### 3.2. mpMRI Data

All patients underwent mpMRI according to the study protocol. Twenty-eight patients had PI-RADS III or higher: twelve in the PROSTAFLOG^®^ group and fourteen in the Serenoa Repens 320 mg group. The mpMRI findings are displayed in Table 2.

### 3.3. PSA Values at the Follow-Up Visit

**Figure 2:**
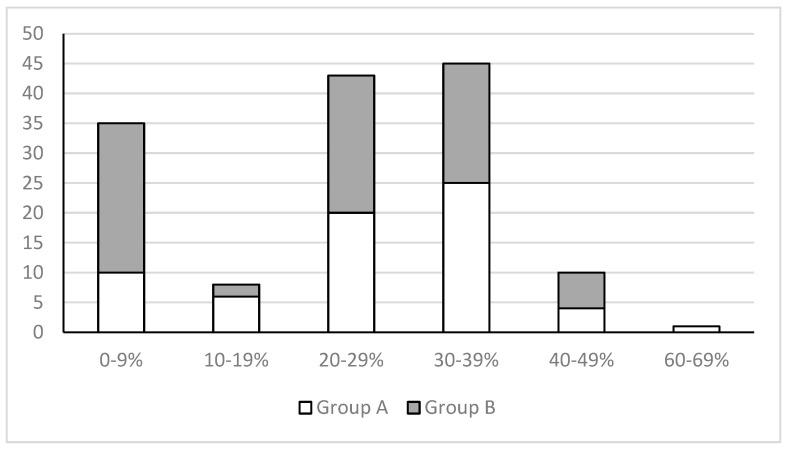
The figure shows the percentage reduction in PSA after three months for both groups. The *x*-axis shows the percentage reduction in PSA, and the *y*-axis shows the number of patients for each group put on top of each other.

Fifty patients in the Prostaflog^®^ group (75.7%) showed a significant reduction in total PSA compared to forty-nine in the Serenoa Repens group (64.4%) (*p* < 0.001) (Figure 2). No significant difference was found between the two groups in terms of free/total PSA ratios. All PSA values are displayed in Table 2.

### 3.4. Decisions on Prostate Biopsy and Findings of Prostate Cancer

All patients with PI-RADS III or higher in mpMRI underwent prostate biopsy. In the Prostaflog^®^ group, 23 patients (34.8%) underwent prostate biopsy based on their mpMRI evaluations and PSA kinetics compared to 59 (77.6%) in the Serenoa Repens group. Overall, prostate cancer was detected in 29 patients in both groups in the histopathological analyses: 13 in the Prostaflog^®^ group (19.7%) and 16 in the Serenoa Repens group (21.0%). Three patients in the Prostaflog^®^ group showed a significant reduction in total PSA values and had positive mpMRI (6%) findings, while nine in the Serenoa Repens group (19.5%) showed positive findings (*p* < 0.001). Moreover, 7 patients in the Prostaflog^®^ group did not show any significant reduction in total PSA values and had negative mpMRI (43%) findings, while 22 in the Serenoa Repens group (81.4%) had negative findings. The ability of PSA reduction after Prostaflog^®^ therapy to predict positive mpMRI findings showed a sensitivity of 75% (CI 42.8–94.5) and a specificity of 87% (CI 75.1–94.6), with a positive likelihood ratio of 5.7 (CI 2.2–12.4) and a negative likelihood ratio of 0.29 (CI 0.11–0.77). All correlations between PSA values and mpMRI findings between the two groups are displayed in Table 3.

## 4. Discussion

### 4.1. Major Findings

Here, we demonstrate for the first time that a three-month course of a combination of Curcuma Longa, Boswellia, Pinus pinaster and Urtica dioica for men with higher-than-normal PSA levels is superior to Serenoa Repens in reducing PSA values. Significantly fewer men in the combination group underwent prostate biopsy based on changes in PSA values and mpMRI findings. Our study demonstrates that Prostaflog^®^ is an interesting non-antibiotic option to enhance the utility of PSA in the decision making before prostate biopsy and might help avoid unnecessary procedures, reducing the risk of infectious complications related to the invasive procedure.

### 4.2. Understanding Study Findings

Several studies have shown that some plant extracts, such as isoflavones, exert a direct effect on the PSA level and prostate volume, both in patients with prostate cancer and with benign prostatic enlargement. This is due to the direct inhibition of 5-alpha-reductase by isoflavones. Engelhardt PF et al. demonstrated that a daily dose of isoflavones caused a 10% reduction in prostate volume after 1 year for patients with benign prostatic enlargement [19]. The difference between Prostaflog^®^ and Serenoa Repens in our study is probably due to the presence of compounds, such as curcuma, boswellia, urtica dioica and pinus pinaster. In particular, boswellia, urtica dioica and soybean extracts have been shown to have a measurable effect on the prostate [10]. In particular, Cai et al. recently demonstrated that Prostaflog^®^ is able to decrease prostate inflammation by affecting the IL-8 level [20]. This means that phytotherapeutic compounds can reduce the PSA level through a hormonal as well as an anti-inflammatory pathway. For Serenoa Repens however, the anti-inflammatory effect seems limited. Furthermore, several authors reported the anti-inflammatory and antioxidant properties of Boswellic acids in combination with other phytotherapeutic compounds, highlighting their therapeutic role in different clinical contexts [21,22]. On the other hand, urtica dioica is able to significantly reduce the level of heat shock protein (HSP) 70, highlighting an important anti-inflammatory and antioxidant effect [23]. The effect of Prostaflog^®^ on the PSA level could be explained by the synergic effect of its compounds, acting on the level of inflammation and through the inhibition of 5-alpha-reductase [21,22], reducing the prostate growth.

### 4.3. Results in Comparison with Other Studies

The positive relationship between prostate inflammation/infection and elevated PSA values is well established [24]. For this reason, many authors have been looking for ways to reduce elevated PSA that might be due to inflammation in order to reduce the number of unnecessary biopsies. With this aim, urologists have prescribed antibiotics for elevated PSA levels as an empirical treatment of subclinical infection or inflammation. This practice finds no support in international guidelines and violates antimicrobial stewardship principles [21,25,26,27]. In 2015, Busato et al. already demonstrated that empirical antibiotic treatment of asymptomatic male patients did not lead to PSA reduction and highlighted the risk of collateral damage in the form of increased antimicrobial resistance [27]. Moreover, they underlined that even a PSA reduction greater than 10% after antibiotic treatment of this population should not postpone prostate biopsy [27]. In the same way, Baltaci et al. reported that 5 out 17 patients who experienced a decreased PSA level to <4 ng/mL had prostate cancer upon biopsy [28]. In 2018, Fabiani et al. performed a prospective study verifying the effect of Prostaflog^®^ on PSA values and evaluating its implications in terms of reducing the number of prostate biopsies performed [29]. In this study, they enrolled 50 patients with PSA values > 4 ng/mL or PSA velocities (PSAv) > 0.75 ng/mL/years. All patients underwent a treatment of two tablets per day of Prostaflog^®^ for 30 days. A follow-up visit was planned after PSA evaluation at the end of the treatment. All patients underwent prostate biopsy in cases of PSA values persistently >4 ng/mL or more. The authors concluded that the use of Prostaflog^®^ is able to reduce the PSA value even if they were not able to know if the reduction in PSA after treatment could exclude a prostate cancer diagnosis [29]. In our paper, we demonstrated that a treatment with Prostaflog^®^ seems to be an interesting tool to avoid unnecessary prostate biopsies for men with higher-than-normal PSA levels. Recently, Cindolo et al. enrolled 100 patients affected by prostatitis-like symptoms in a multicentric phase III study [30]. All patients underwent a treatment of one tablet per day of Prostaflog^®^ for 60 days. In this study, the authors demonstrated a significant inflammation index reduction and PSA value. They concluded that Prostaflog^®^ represents a promising and safe therapeutic compound, leading to a significant reduction in symptoms, inflammation markers in chronic prostatitis and benign prostatic hyperplasia [30]. On the basis of these considerations, a combination of Curcuma Longa, Boswellia, Pinus pinaster and Urtica dioica shows significant anti-inflammatory properties that are able to reduce prostate inflammation and reduce PSA levels without altering the diagnosis of prostate cancer.

### 4.4. Clinical Implications

No other studies have so far have explored the role of phytotherapy for patients with elevated PSA before prostate biopsy. We found that only three patients in the Prostaflog^®^ group showed a significant reduction in total PSA value while having positive mpMRI findings (6%), compared with nine in the Serenoa Repens group (19.5%) (*p* < 0.001), thereby showing that the risk of lowering PSA caused by prostate cancer by means of a hormonal pathway is very low. After the introduction of mpMRI, the number of unnecessary prostate biopsies has decreased and the role of PSA as diagnostic marker of prostate cancer has been reduced [2,3,31]. However, the use of mpMRI is not yet widespread in all countries, and we therefore still need tools that enable us to differentiate if an elevated PSA is due to prostate cancer or inflammation [32]. Moreover, the use of mpMRI is also associated with high costs. In this sense, the use of tools such as Prostaflog^®^ may help us to differentiate if an elevated PSA is due to prostate cancer or inflammation.

### 4.5. Strengths and Limitations of the Present Study

A strength of this study is that a three-month experimental study of a PSA-lowering treatment could be performed in a country where it did not violate ethical or public health regulations. The study reports real-life practice that was not changed by the protocol. Another strength is that the contents of the phytotherapeutic compounds studied are well known. In fact, the use of the phytotherapeutic compounds tested in this study has been supported by some previously published studies evaluating the role of Prostaflog^®^ in the management of prostate disease [29,30]. Moreover, the use of an antimicrobial-sparing approach for patients with increased PSA values should be considered a strength of this study. It is very important, particularly in the era of antimicrobial resistances. The weaknesses of the study are related to the low number of patients, the short follow-up period, the diagnostic uncertainties of the Prostate Imaging Reporting and Data System (PIRADs) classification, the representativity of the prostate biopsies performed and the lack of long-term observational data, especially regarding PSA development and results of repeat prostate biopsies. Finally, the fact that all enrolled patients were unblinded should be considered a limitation of the study.

## 5. Conclusions

In conclusion, a combination of Curcuma Longa, Boswellia, Pinus pinaster and Urtica dioica given to men with higher-than-normal PSA levels for three months was superior to Serenoa Repens in reducing PSA values. After treatment, significantly fewer men in the combination group underwent prostate biopsy based on changes in PSA values and mpMRI findings. Phytotherapy seems to be an interesting tool to avoid unnecessary prostate biopsies for men with higher-than-normal PSA levels. Prostaflog^®^ might be a good alternative for urologists who believe that an elevated PSA might be caused by inflammation and who prefer an antibiotic-sparing approach as a diagnostic tool before performing prostate biopsy. Further studies with longer follow-up periods and with higher numbers of enrolled patients are needed to confirm our data.

## Figures and Tables

**Table 1:** The table shows demographic, anamnestic, clinical and laboratory data of all patients at enrollment.

	Treatment Group A	Control Group B	*p*
No. of patients	66	76	
Age (years)			0.97
Median (IQR ^†^)	65 (56–76)	66 (54–77)	
BMI (Kg/m^2^)			0.71
Median (IQR ^†^)	27 (25–31)	28 (26–30)	
Prostate volume (cc)			0.11
Median (IQR ^†^)	52 (41–87)	54 (42–86)	
Charlson Comorbidity Index (CCI)			0.08
0	51 (77.2)	57 (75.0)	
1	15 (22.8)	19 (25.0)	
2	0 (-)	0 (-)	
Urinary symptoms			0.10
Voiding	12 (18.2)	15 (19.8)	
Storage	4 (6.0)	5 (6.5)	
Mixed	15 (22.7)	17 (22.3)	
Absence of symptoms	35 (53.1)	39 (51.4)	
PSA total (ng/mL)			0.52
Median (IQR ^†^)	6.7 (4.1–8.9)	6.5 (4.3–9.0)	
PSA free/total (%)			0.90
Median (IQR ^†^)	12 (8–14)	11 (8–13)	

BMI = Body Mass Index; IQR ^†^ = interquartile range; cc = cubic centimeter; PSA = prostate-specific antigen.

**Table 2:** The table shows all multiparametric magnetic resonance imaging findings and PSA values at the follow-up visit.

	Treatment Group A	Control Group B
No. of patients	66	76
mpMRI		
PI-RADS I	10 (15.1)	12 (15.8)
PI-RADS II	44 (66.6)	50 (65.7)
PI-RADS III	9 (13.6)	9 (11.9)
PI-RADS IV	2 (3.1)	4 (5.3)
PI-RADS V	1 (1.6)	1 (1.3)
PSA total (ng/mL)		
Median (IQR ^†^)	3.1 (1.8–5.1)	4.2 (2.1–8.4)
PSA free/total (%)		
Median (IQR ^†^)	14 (10–17)	12 (9–15)
PSA density (ng/mL^2^)		
Median (IQR ^†^)	0.13 (0.11–0.16)	0.13 (0.12–0.16)

mpMRI = multiparametric magnetic resonance imaging; PSA = prostate-specific antigen; IQR ^†^ = interquartile range.

**Table 3:** The table shows correlations between changes in PSA values and multiparametric magnetic resonance imaging findings in the two groups.

	Treatment Group A		Control Group B	
No. of patients	66		76	
	ΔPSA (−)	ΔPSA (+)	ΔPSA (−)	ΔPSA (+)
mpMRI (PI-RADS III or higher)	3	9	9	5
mpMRI (PI-RADS I or II)	47	7	40	22

mpMRI = multiparametric magnetic resonance imaging; ΔPSA (−) = clinically significant reduction in PSA between enrollment and control; ΔPSA (+) = not clinically significant reduction in PSA between enrollment and control.

## Data Availability

Data are unavailable due to privacy or ethical restrictions in accordance with Italian bylaws.
